# Supplementary material for: Revealing exciton masses and dielectric properties of monolayer semiconductors with high magnetic fields
Source: Nat Commun. 2019 Sep 13;10:4172. doi: 10.1038/s41467-019-12180-y (PMC6744484; doi:10.1038/s41467-019-12180-y)
Supplement: Supplementary file 1 — Supplementary Information [file 41467_2019_12180_MOESM1_ESM.pdf]

Revealing exciton masses and dielectric properties of monolayer semiconductors with high magnetic fields

M. Goryca *et al.*

SUPPLEMENTARY INFORMATION

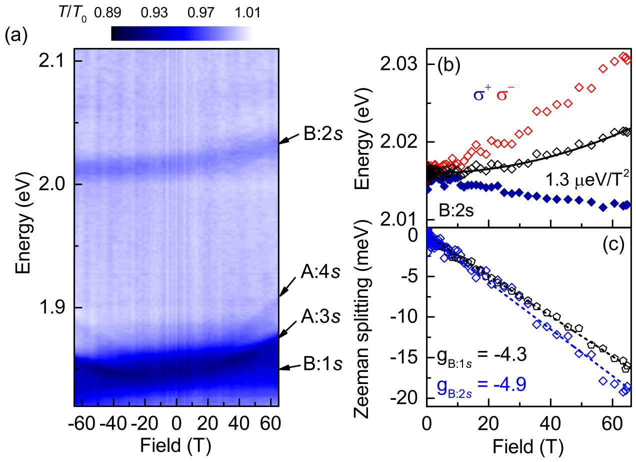

Supplementary Figure 1. Magneto-optical spectroscopy of the B exciton in monolayer $MoSe_2$. (a) Normalized intensity map of the field-dependent transmission spectra through monolayer $MoSe_2$ in the energy range around the excited B:2s exciton. (b) Energies of the $\sigma^{\pm}$ polarized B:2s exciton, and their averaged value (black symbols). The black line is a quadratic fit, indicating a diamagnetic shift coefficient 1.3 μeV T^{-2} for the B:2s exciton. (c) The valley Zeeman splitting of the B:1s and B:2s exciton states; dashed lines show linear fits.

Owing to the excellent optical quality of the $MoSe_2$ structures it was possible to observe and track the excited 2s state of the higher-energy B exciton. Supplementary Figure 1 shows that the B:2s absorption line is visible about 167 meV above the B:1s exciton, and therefore the 1s–2s energy separation is very similar to that observed for the A exciton. In magnetic field the B:2s exciton evinces a strong valley Zeeman splitting with effective g-factor $g \approx -4.9$, as well as a clearly discernible quadratic diamagnetic shift of 1.3 μeV T^{-2} that is comparable to the value calculated for the A:2s state.

Unfortunately the B:1s peak is too broad and weak and its diamagnetic shift is too small to measure accurately. Such a measurement of the B:1s diamagnetic shift is further hindered by its overlap with the A:3s absorption line and also by the proximity to the A:2s state, which makes accurate fitting unreliable. Therefore we can quantitatively and accurately compare only the diamagnetic shifts of B:2s state and the A:2s state. The similarity between those shifts suggests similar effective masses for the A and B excitons, which contradicts general expectations that B excitons have larger mass; however, as discussed in detail in the main text, the actual mass of the A exciton significantly exceeds predicted values. Clearly the effective mass analysis deserves further work in both experiment and theory in the future in the high quality and tunable samples that are now available.
